# Commentary—District decision-making to strengthen maternal, newborn and child health services in low-income settings

**DOI:** 10.1093/heapol/czw103

**Published:** 2016-09-01

**Authors:** Kara Hanson, Joanna Schellenberg

**Affiliations:** London School of Hygiene and Tropical Medicine, UK

**Keywords:** Decision making, health services, health systems research, health information system, maternal and child health

Information systems and health planning are relatively neglected areas of health policy and system research. A rapid search of papers published in *Health Policy and Planning* identified only 15 out of a total of 726 papers published the last 5 years on the topics of information systems, monitoring and evidence-informed decision-making. Some of these papers focused on the extent to which research evidence is used by policymakers and health system managers, yet collecting high quality routine information and using this information to plan and evaluate health services is critical for health system strengthening.

The articles in this series outline a model for a data-informed platform for health which would bring together routine information from the public and private sectors on health care inputs and processes including service delivery, that could inform decision making, priority setting and planning at the district level, and assist in the evaluation of maternal, newborn and child health (MNCH) services. This is a useful extension to the literature on health information systems because of its focus on how information is used to take decisions at the local level. By keeping data users at the centre of the system, it raises important questions of who collects information and how it flows among levels of the system—all of which influence the incentives to generate valid data on health services, their coverage and key inputs used in delivering them.

This question of whose needs health information should serve was at the centre of the research presented in these articles. The work emerged from a group working on improving routine government programmes on maternal and child health in three challenging settings: North-East Nigeria, Ethiopia, and the state of Uttar Pradesh in India, all of which have relatively large population sizes and poor health outcomes. The research team were originally interested in developing measures of implementation strength (Hargreaves *et al.* 2016) in each district, and in how these might be used to evaluate programmes across several districts. They approached local health leaders employed by government in each of these settings with this in mind and quickly found that these local health leaders had a strong interest in improving their own use of their own data for their own decisions. The concept of the ‘data-informed platform for health' was created in response, to empower local health teams to use their own data in a structured and systematic way.

The information collected in many systems is partial, missing important contributions to health. One innovation of the data-informed platform for health is its focus on the whole health system, including the private sector, which is an important provider of services in many settings; and in including non-health sectors, such as the department of women and child development in India. To optimize the use of public resources in mixed health systems, it is vital to understand the activities of all the actors that are involved in producing health, bringing a vision of health that is aligned with a social determinants perspective and with multi-sectoral targets such as the sustainable development goals.

The first paper in this series (Avan *et al.* 2016) defines the nascent concept of a data-informed platform for health, which is a structured and standardised process for local health teams to make use of existing data to plan, track, review and course-correct their programmes. In a feasibility study of this approach in a district health systems context in five districts across the three countries, the researchers identified multiple barriers to the use of local data for health decisions at district level, and no standardised processes. Of the three settings, India had the most amenable context: the Indian state of West Bengal is the focus of a pilot phase in 2015–16.

The second paper (Wickremasinghe *et al.* 2016) is a systematic literature review about processes to support the use of health data in decision-making at district level in low-income settings. Despite including grey literature in the search only 14 reports were identified. This illustrates the challenges of relying on literature reviews for complex topics in health system strengthening, in which search terms are frequently non-specific. However, it also points to the more general problem that much of the accumulated experience and knowledge about how to strengthen the collection and use of routine health information has been generated by projects and programmes that have not documented their lessons in a systematic or accessible way, making it difficult for others to learn from their experience. Tapping into the tacit knowledge of those involved in system strengthening may offer greater opportunities for sharing approaches, successes and challenges.

The third paper (Bhattacharyya *et al.* 2016) is a case study in Ethiopia and India showing untapped potential of Health Management Information System (HMIS) data for MNCH decision-making at district level. The authors documented a surprisingly large number of HMIS data elements in both settings: over 11 000 in India, and over 4000 in Ethiopia, including aspects of service delivery, medical supplies, workforce, governance and finance. In both countries they identified an existing district-level platform that brings public and private sectors together. Although formal data sharing between public and private health sectors was minimal, these existing platforms give an opportunity for improved use of district-level data for decision-making through the data-informed platform for health approach.

The fourth paper (Gautham *et al.* 2016) presents prospects for engaging the private sector in health data sharing and collaborative decision-making at district level in India. It reminds us of the diversity of the private sector in many settings, which includes large formal providers with health and management information systems that support their own decision making, small sole practitioners, and range of ‘less than fully qualified’ providers (Berman 2000) who may be acting outside the regulatory system and reluctant to engage in initiatives which might constrain their practices. The article finds considerable willingness to engage in a data-informed platform for health among parts of the sector, but that this varies because of differences in capabilities, incentives and the level of trust between providers and the government.

From the set of papers, we can see some of the elements of what a data-informed platform for health would look like, and some valuable suggestions about sequencing issues and constraints that need to be addressed before such a system would be feasible. But even in Uttar Pradesh, which according to the TELOS framework is in the most advanced state of readiness for a data-informed platform for health, there is no strong sense of how to harness the latent demand for better information and better decision making. It will be critical that any new system is able to fit into the complex set of activities, meetings and reporting requirements that frontline district managers confront every day. After an initial start-up and testing phase, sustainability will depend on both district and state-wide endorsement, engagement and ownership. The more integrated approach to information is welcome, but in moving towards implementation it will be important to consider the insights of the broader health systems literature which identifies the complex nature of systems, and the mechanisms through which innovations are adopted and spread. Most important will be to involve those who will be responsible for operating the system in its design and implementation.

*Conflict of interest statement*. None declared.
